# A multicentre case control study on complicated coeliac disease: two different patterns of natural history, two different prognoses

**DOI:** 10.1186/1471-230X-14-139

**Published:** 2014-08-07

**Authors:** Federico Biagi, Alessandra Marchese, Francesca Ferretti, Rachele Ciccocioppo, Annalisa Schiepatti, Umberto Volta, Giacomo Caio, Carolina Ciacci, Fabiana Zingone, Anna D’Odorico, Antonio Carroccio, Giuseppe Ambrosiano, Pasquale Mansueto, Antonio Gasbarrini, Anna Chiara Piscaglia, Alida Andrealli, Marco Astegiano, Sergio Segato, Matteo Neri, Alberto Meggio, Giovanni de Pretis, Italo De Vitis, Paolo Gobbi, Gino Roberto Corazza

**Affiliations:** 1 Coeliac Centre/Department of Clinical Medicine, St Orsola-Malpighi University Hospital, Bologna, Italy; 2 Department of Medicine and Surgery, Gastrointestinal Immune Diseases Centre, University of Salerno, Salerno, Italy; 3 Department of Surgical and Gastroenterological Sciences, University of Padua, Padua, Italy; 4 Ospedali Civili Riuniti di Sciacca, University of Palermo, Palermo, Italy; 5 Internal Medicine, University of Palermo, Palermo, Italy; 6 Department of Internal Medicine, Catholic University of Sacred Heart, Gemelli University Hospital, Rome, Italy; 7 Department of Gastro-Hepatology, AOU San Giovanni Battista Molinette, University of Turin, Turin, Italy; 8 Gastroenterology and Endoscopy Unit, Ospedale di Circolo di Varese, Fondazione Macchi, Turin, Italy; 9 Department of Internal Medicine, University “G D'Annunzio” of Chieti, Turin, Italy; 10 UO Multizonale di Gastroenterologia ed Endoscopia APSS Trento, Rome, Italy; 11 Internal Medicine and Gastroenterology Unit, UCSC-CIC, Rome, Italy; 12 Coeliac Centre/First Department of Internal Medicine, Fondazione IRCCS Policlinico San Matteo, University of Pavia, P.le Golgi, 19, I-27100, Pavia, Italy

**Keywords:** Celiac disease, Complications, EATL, Prognosis, Glutens, Gluten-free diet

## Abstract

**Background:**

Coeliac disease is a common enteropathy characterized by an increased mortality mainly due to its complications. The natural history of complicated coeliac disease is characterised by two different types of course: patients with a new diagnosis of coeliac disease that do not improve despite a strict gluten-free diet (type A cases) and previously diagnosed coeliac patients that initially improved on a gluten-free diet but then relapsed despite a strict diet (type B cases). Our aim was to study the prognosis and survival of A and B cases.

**Methods:**

Clinical and laboratory data from coeliac patients who later developed complications (A and B cases) and sex- and age-matched coeliac patients who normally responded to a gluten-free diet (controls) were collected among 11 Italian centres.

**Results:**

87 cases and 136 controls were enrolled. Complications tended to occur rapidly after the diagnosis of coeliac disease and cumulative survival dropped in the first months after diagnosis of complicated coeliac disease. Thirty-seven cases died (30/59 in group A, 7/28 in group B). Type B cases presented an increased survival rate compared to A cases.

**Conclusions:**

Complicated coeliac disease is an extremely serious condition with a high mortality and a short survival. Survival depends on the type of natural history.

## Background

Coeliac disease (CD), a chronic gluten-induced enteropathy, is common in western populations [1]. Although its prognosis is excellent in the great majority of cases, some of these patients may develop serious complications, such as refractory CD type 1 (RCD1), refractory CD type 2 (RCD2), ulcerative jejuno-ileitis (UJI), enteropathy associated T cell lymphoma (EATL), abdominal B cell lymphoma (ABL), small bowel carcinoma (SBC) and collagenous sprue (CS) [2-6]. Since treatment is hardly effective [7], they drastically reduce the prognosis of these patients [2]. In particular, five-year survival is between 80% and 96% in patients with RCD1, between 40% and 58% in patients with RCD2 and drops to less than 20% in patients with CD complicated by EATL [8-13]. These conditions have traditionally been considered the major complications of CD [14]. Moreover, since most of them share a common pathogenetic link [15], it is certainly possible to consider them altogether as complicated CD (CCD).

There are various factors correlated with the risk of developing CD complications. It has been known for some time that the main risk factor is poor compliance with a gluten-free diet (GFD) and, in the last few years, it has emerged that the clinical form of CD at diagnosis (classic/major, non-classic/minor, asymptomatic/silent [16]), the diagnostic delay of CD, age at diagnosis of CD, and homozygosity for HLA-DQ2 also correlate with the risk of developing complications and thus with mortality [17-23]. Despite this additional knowledge, there are still many obscure aspects.

The natural history of CCD has not yet been the subject of specifically focussed studies. According to our experience, there are two different ways in which CCD can develop clinically [24]. In some patients, the complication of CD is diagnosed shortly after the diagnosis of CD itself and the introduction of a strict GFD does not result in a significant clinical improvement (for the purpose of this paper we define these patients as “type A cases”). Conversely, in other patients the diagnosis of CCD is made after a period of time during which the introduction of a strict GFD is followed by a remission of the symptoms that had led to the initial diagnosis of CD. Subsequently, however, the reappearance of malabsorption, abdominal pain, fever, or anaemia arouses the suspicion that a complicated form of CD is developing (“type B cases”). The prevalence and, above all, the prognosis of these two different forms of onset of CCD have not yet been ascertained.

The aim of this study was to investigate the natural history of CCD and to clarify its evolution and prognosis.

## Patients and methods

### Design of the study

To collect a sufficient number of patients, we organised a retrospective multicenter case–control study based on collection of clinical and laboratory data.

### Cases and controls

Cases were recruited among adult coeliac patients (age > 18 years), who, between 1990 and 2010, were diagnosed with any of the following complications: RCD1, RCD2, UJI, EATL, ABL, SBC, CS. Diagnoses of EATL, ABL, SBC, and CS were based on morphological criteria; diagnosis of RCD2 was based on a flat duodenal mucosa not responding to 12 months on a GFD and evidence of an aberrant intraepithelial lymphocyte population and/or gamma-chain T-cell monoclonal rearrangement; diagnosis of RCD1 was based on a flat duodenal mucosa not responding to 12 months on a GFD but without the diagnostic criteria for RCD2; diagnosis of UJI was based on the demonstration of small bowel ulcers but without the diagnostic criteria for EATL and RCD2 [3,9-12]. Cases were divided into types A and B according to the type of natural history (see introduction). Other causes of villous atrophy or clinically nonresponsive CD were excluded [25,26].

Controls were recruited among adult patients (age > 18 years) found to be affected by CD on the basis of a flat duodenal biopsy and positive endomysial/tissue transglutaminase antibodies while on a gluten-containing diet. They all clinically responded to a GFD and none of them developed any of the above mentioned complications. Two cases, or at least one, were matched to cases by gender, age at diagnosis of CD (±5 years), year of first observation by the cooperating centre and length of follow-up.

### Data collection

Date of birth, gender, date of diagnosis of CD, pattern of clinical presentation of CD (classic, non classic, asymptomatic) [16], adherence to a GFD evaluated by means of a dietary interview, clinical response to a GFD, date of diagnosis of CCD, type of complication, date and cause of death (if the subject is still alive, date of the last time he/she was seen in the clinic) are the very simple clinical data we collected for each case and each control. Haemoglobin, RDW, albumin, Na^+^, K^+^, Ca^++^, total cholesterol, triglycerides, glucose, ESR, and CRP, at the time of diagnosis of CD, are the laboratory tests we evaluated in cases and controls.

### Statistics

The data obtained were analysed by means of descriptive statistics, Student’s T test, and Mann–Whitney test where appropriate. To study the survival over time of patients with CCD we calculated the cumulative probability curve by means of the Kaplan-Meier method. The complication-free survival was calculated from the date of diagnosis of CD to that of diagnosis of complication, whereas the survival of the CCD patients were calculated from the date of diagnosis of complication to that of last known vital status. The logrank analysis, performed according to the Mantel-Cox method, and evaluated the presence of any differences between the curves obtained [27,28].

The study protocol conforms to the ethical guidelines of the 1975 Declaration of Helsinki (6th revision, 2008) as reflected in a priori approval by the institution's human research committee (Fondazione IRCCS Policlinico San Matteo).

## Results

### Clinical study

Eleven Italian centres, distributed throughout the country, took part in the study, allowing us to collect the clinical data of 87 cases (55 females, mean age at diagnosis of CD 51.2 ± 14.3 years, median follow-up from the diagnosis of CD 6.4 years, range 0.1-30.3) and 136 controls (89 females, 49.2 ± 13.3 years). Twenty of them were affected by RCD1, 3 by RCD2, 17 UJI, 17 EATL, 6 ABL, 16 SBC, and 1 CS. In seven patients with RCD, the available data did not make it possible to distinguish between RCD1 and RCD2 (RCDX).

**Figure 1:**
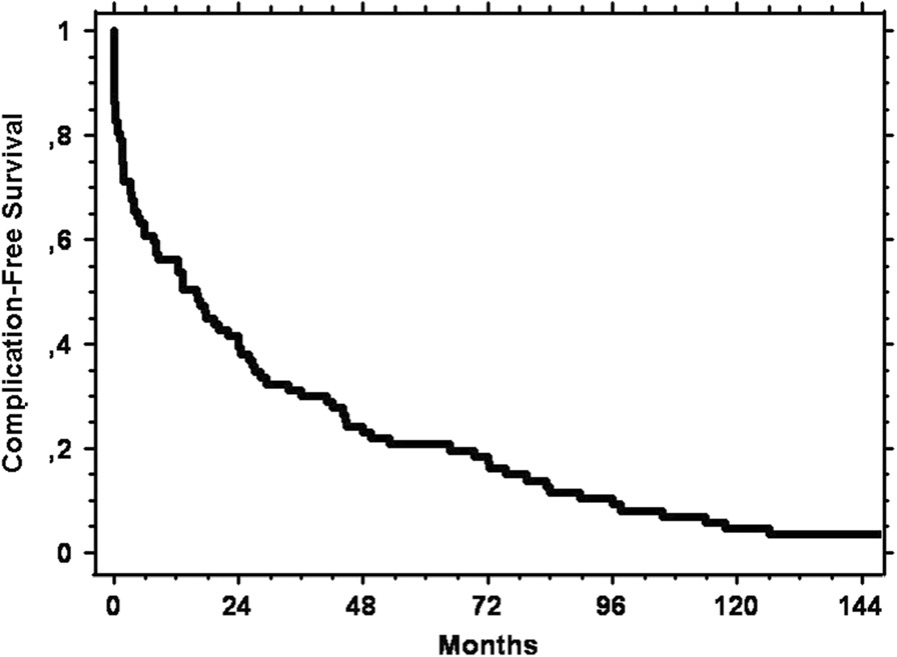
Kaplan Meier curve showing the cumulative complication-free survival from diagnosis of coeliac disease until diagnosis of complicated coeliac disease in the 87 cases.

Table 1 shows the main clinical results. As expected, clinical type of CD, adherence to a GFD and mortality were significantly different in the two groups. Figure 1 shows the Kaplan Meier curve calculated on the basis of the complication-free time. Although most cases developed the complication soon after the diagnosis of CD, there is no limit beyond which the complications of CD no longer develop.

**Table 1:** Demographic and clinical data in complicated coeliac disease (cases) and in coeliac patients responding to a gluten-free diet (controls)

	**Cases**	**Controls**	**p**
**Number (Females)**	87 (55)	136 (89)	NS*
**Age at diagnosis of CD (mean year ± sd)**	51 ± 14	49 ± 13	NS**
**Age at diagnosis of CCD (mean year ± sd)**	54 ± 13	N.A.	
**Classic form at diagnosis of CD**	86/87 (99%)	85/136 (63%)	<0.0001*
**Strict adherence to a gluten-free diet**	66/87 (76%)	129/136 (95%)	<0.0001*
**Years between diagnosis of CD and diagnosis of CCD: median (25°, 75° percentile)**	1.4 (0.1, 3.8)	N.A.	
**Mortality**	37/87 (43%)	0/136 (0%)	<0.0001*
**Years between diagnosis of CCD and death: median (25°, 75° percentile)**	1.0 (0.1, 2.9)	N.A.	

*Chi^2^ test, **t test, CD = coeliac disease; CCD = complicated coeliac disease; N.A. = not allowed; NS = not significant.

Table 2 show the clinical data of the cases divided according to the type of natural history. In 59 cases (68%) the complication arose without GFD, introduced after the initial diagnosis of CD based on flat duodenal biopsy, positive coeliac antibodies and/or coeliac HLA, having led to a considerable improvement in symptoms (type A cases). EATL affected 16 patients, UJI 13, SBC 12, RCD1 8, RCD2 1, RCDX 4, and ABL 5. Only 28 of them (47%) were still alive when these data were collected. Apart from five cases who died of cardiovascular diseases (3), Paget disease (1), and lung cancer (1), the other cases all died because of the condition complicating CD.

**Table 2:** Demographic and clinical data in the two forms of complicated coeliac disease: type A cases, coeliac patients who never responded to a gluten-free diet, type B cases, coeliac patients who initially responded to a gluten-free diet but then relapsed

	**Cases type A**	**Cases type B**	**p**
**Number (Females)**	59 (32)	28 (23)	0.02*
**Age at diagnosis of CD (mean years ± sd)**	53 ± 14	47 ± 14	NS**
**Age at diagnosis of CCD (mean year ± sd)**	54 ± 13	54 ± 13	NS**
**Strict adherence to a gluten-free diet**	45/59 (83%)	21/28 (75%)	NS*
**Years between diagnosis of CD and diagnosis of CCD: median (25**^**th**^**, 75**^**th **^**percentile)**	0.3 (0.1, 1.4)	4.9 (3.3, 8.1)	<0.0001***
**Mortality**	30/59 (51%)	7/28 (25%)	0.03*
**Years between diagnosis of CCD and death: median (25**^**th**^**, 75**^**th **^**percentile)**	1.0 (0.1, 2.7)	2.3 (0.3, 4.2)	NS***

*Chi^2^ test, **t test, ***Mann–Whitney test, CD = coeliac disease; CCD = complicated coeliac disease; NS = not significant.

In the last 28 cases (32%), a diagnosis of CCD was reached after a period of time (median 5.0 years, 25^th^-75^th^ percentile 3.3-8.1 years) in which the GFD had led to a remission of the malabsorption symptoms that had triggered the diagnosis of CD (cases type B). RCD1 was diagnosed in 12 patients, RCD2 in 2, RCDX in 3, UJI in 4, SBC in 4, EATL, ABL, and CS in one patient each. Twenty-one out of 28 (75%) were still alive at the time when these data were collected; the other cases all died because of the condition complicating CD.

Table 3 shows the main laboratory test results at the time of diagnosis of CD in cases and controls. Already at the time of diagnosis of CD, levels of haemoglobin, albumin, cholesterol and serum electrolytes were lower than in controls. Conversely, inflammatory markers were higher than in controls.

**Table 3:** Mean ± standards deviation of the laboratory findings in patients with complicated coeliac disease (cases) and in coeliac patients responding to a gluten-free diet (controls)

	**Cases**	**Controls**	**p**
**Haemoglobin (g/dL)**	11.0 ± 2.1	12.0 ± 2.2	0.0038
**RDW (%)**	13.8 ± 2.0	15.4 ± 6.9	NS
ESR* (mm/h)	20 (9–35)	10 (6.8-15)	0.005
CRP* (mg/dL)	0.9 (0.5-1.9)	0.4 (0.3-0.5)	< 0.0001
**Albumin (g/dL)**	3.2 ± 0.7	4.1 ± 0.6	0.0001
**Na**^**+ **^**(mEq/L)**	137.9 ± 6.9	141.1 ± 3.5	0.03
**K**^**+ **^**(mEq/L)**	4.1 ± 0.7	4.3 ± 0.4	0.02
**Ca**^**++ **^**(mEq/L)**	8.2 ± 1.2	9.2 ± 0.5	< 0.0001
**Total cholesterol (mg/dL)**	145.9 ± 39.7	183.3 ± 45.7	< 0.0001
**Triglycerides (mg/dL)**	110.3 ± 61.3	102.5 ± 46.7	NS
**Glucose (mg/dL)**	90.0 ± 14.8	89.7 ± 18.8	NS

*median and 25^th^-75^th^ inter-quartile range.

As far as possible differences between type A and type B cases are concerned, there was no difference between these two groups in any of the laboratory tests, apart from triglyceride levels that were higher in type A cases (133.5 mg/dL ± 74.8) than in type B cases (87.0 mg/dL ± 31.4).

### Survival study

**Figure 2:**
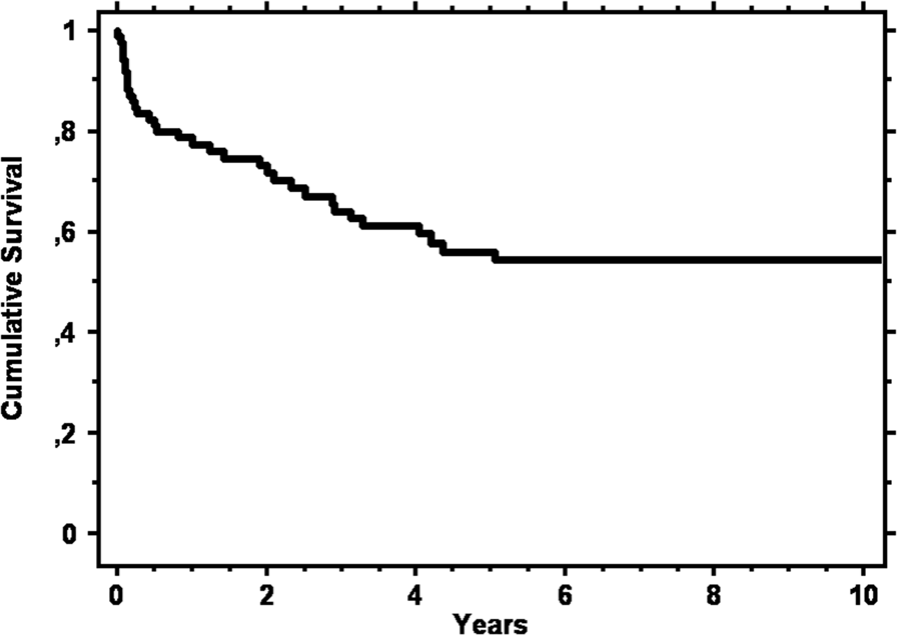
Kaplan Meier curve showing the cumulative survival from diagnosis of complicated coeliac disease until time of death in the 87 cases.

**Figure 3:**
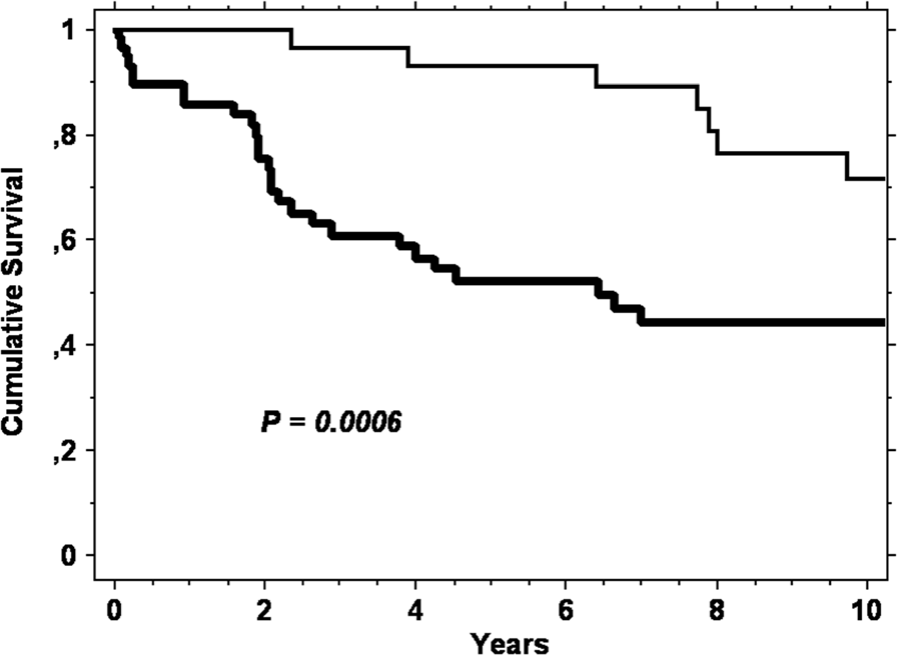
**Kaplan Meier curve showing the cumulative survival from diagnosis of complicated coeliac disease until time of death in the 87 cases divided according to the type of natural history of disease.** Thin line: type B cases, who initially responded to a gluten-free diet; thick line: type A cases, who never responded to a strict gluten-free diet.

The mortality of this condition is extremely high, since only 50 out of the 87 cases (58%) were still alive when these data were collected. Figure 2 shows the cumulative survival of the 87 cases, starting from the time at which CCD was diagnosed. It can be observed that survival was drastically reduced in the first 6 months and continued to be reduced, albeit more slowly, up to 5 years after the diagnosis of CCD. Once this limit has been passed, the survival seems to stabilize.Figure 3 shows the cumulative survival at 10 years according to the two different types of natural history. Type B cases presented an increased survival rate compared to type A cases (logrank, p = 0.0006).

Cumulative 10-year survival according to the histopathological subtype of CCD, analysed by means of Kaplan Meyer curves and subsequent logrank analysis, did not show any statistically significant differences (data not shown). It is, however, likely that this could be due to the fact that it was necessary to divide the cases into six subgroups, with an inevitable reduction of statistical power.

## Discussion

This study investigates the natural history of CCD and provides both new results and confirmation of results already reported in the literature.

Although the complications of CD tend to develop just after the diagnosis of CD in most cases, these complications can also arise even after many years (Figure 1). This confirms the well-known importance of maintaining a regular clinical follow up in all coeliac patients, especially those diagnosed in adulthood. Very interestingly, we demonstrated that on the basis of the initial response to a GFD it is possible to identify two different clinical forms of CCD and that these present substantial differences in terms of survival (Figure 3). In patients who develop a complication after remission of the symptoms that led to the initial diagnosis of CD (type B cases), the prognosis is better than in patients in whom the complication of CD developed without the initial symptoms ever going into remission (type A cases). To explain this finding, we noted that EATL, UJI and SBC are more frequent among cases type A than cases type B. On the other hand, RCD1 is more frequent among cases type B. Thus, we could hypothesise that, in those coeliac patients who developed complications very rapidly without GFD being able to induce any remission of the symptoms, a malignant complication had already been triggered when CD was diagnosed. The course of the disease was therefore much more aggressive. Finally, since nowadays there is no efficient treatment for these complications [7], this finding is unlikely to have an immediate, strong clinical impact. Nevertheless, it certainly helps in better understanding the clinical course of these conditions and in figuring out their prognoses.

As regards the different histopathological subtypes, we showed that RCD, UJI, and EATL, the complications well-known to share a common pathogenetic link [15], are definitely the most common complications (64/87, 73.5%), SBC and ABL occur in 18% and 7% of the cases respectively, and CS seems to be exceptionally rare. Having found that almost 75% of the cases share a common pathogenetic link and that the others are affected by abdominal malignancies certainly supports are choice of considering all the cases as affected by CCD. According to our data, survival in each of these subtypes does not appear to be significantly different. However, since the literature reports very different survival rates for each of these subtypes of complication [8-12], we believe that our result is due to the relatively limited size of our sample rather than to a really similar mortality.

Patients with RCD2 have already been found to be significantly more anaemic and hypoalbuminemic than patients with RCD1 [29]. Here we show that, already at the time of diagnosis of CD, not only haemoglobin and albumin but also electrolytes and cholesterol are significantly reduced in coeliac patients who will later develop complications compared to coeliac patients who will respond to a GFD. The reason why these nutritional parameters are lower in the first ones is likely to be linked to the fact that they are almost always affected by a classic/major form of CD (Table 1). On the other hand, the increased levels of ESR and CRP we found in some cases suggest that an increased systemic inflammatory response, likely to be linked to the onset of the complication, was already present at the time of diagnosis of CD.

Our study made it possible to confirm a series of results already present in the literature, and this is an obvious demonstration of the validity of all our results. In particular, we confirm that the complications of CD are burdened by a high rate of mortality and that death occurs soon after the diagnosis of the complication (Figure 2). We also confirmed that coeliac patients who do not adhere to a strict GFD, with symptoms of classic CD at the onset and with a diagnosis late in life are at greater risk of developing these complications [18].

This study also presents some problems, linked fundamentally to its retrospective and multicentre nature. The study is based on 87 cases who had been attending 11 different Italian centres over the last 15 years; 37 of them were already dead when the data were collected. Moreover, the diagnoses of the CCD subtypes were those made by the individual centres and it was not possible to bring them into line. While this is probably not a problem as regards the diagnosis of lymphoma and cancer of the small intestine, it is inevitable that it influenced the differential diagnosis between RCD1 and RCD2, a diagnosis that has only been possible for a few years and certainly not in all centres. This type of problem is, on the other hand, well known and already reported in the literature [5]. Moreover, in seven of our cases of RCD the available data did not make it possible to distinguish between type 1 and 2. Evaluation of the strictness of a GFD was also necessarily based on the clinical judgement of each centre, without it being possible to even out this evaluation. Finally, our median follow up was rather short (2,9 years). However, this was due to the rapid onset of most complications and to their severe prognosis.

These organisational problems could be resolved with a prospective study. This is, however, very difficult to organise. Since CCD develops in less than 1% of patients [4-6,8], then to enrol at least 100 patients with CCD we would have to diagnose at least 10000 new adult patients with CD, follow them for several years before identifying those that will develop a complication and then study their clinical evolution and survival.

## Conclusions

Our study reveals previously unrecognised, interesting and innovative aspects of CCD. While on one hand we have in fact confirmed a series of data already present in the literature, on the other we have identified two clinical subtypes of CCD distinguished on the basis of the initial clinical response to a GFD and on survival.

## Abbreviations

CD: Coeliac disease; RCD1: Refractory CD type 1; RCD2: Refractory CD type 2; UJI: Ulcerative jejuno-ileitis; EATL: Enteropathy associated T cell lymphoma; ABL: Abdominal B cell lymphoma; SBC: Small bowel carcinoma; CS: Collagenous sprue; GFD: Gluten-free diet.

## Competing interests

The authors declare that they have no competing interests.

## Authors’ contributions

FB planned the study and wrote the manuscript together with FF and GRC; AM, RC, AS, UV,GC, CC, FZ, ADO, AC, GA, PM, AG, ACP, AA, MA, SS, MN, AM, GdP, and IDV took care of the patients and collected the data in the respective centres; FB, FF and PG performed the statistical analysis. All authors read and approved the final manuscript.

## Pre-publication history

The pre-publication history for this paper can be accessed here:

http://www.biomedcentral.com/1471-230X/14/139/prepub
